# Renal cell carcinoma presents as portal vein thrombosis, a very rare combination

**DOI:** 10.1002/ccr3.9220

**Published:** 2025-01-31

**Authors:** Raad Alhaj Tahtouh, Raidah Sawan, Wisam Alwassiti, Ahmad Ali, Khaled Alkhodari, Khalid Azhar

**Affiliations:** ^1^ Internal Medicine Department Hamad Medical Corporation (HMC) Doha Qatar; ^2^ Internal Medicine Department Damascus University Damascus Syria

**Keywords:** comprehensive evaluation, diagnosis, malignancy, portal vein thrombosis, rare complication, renal cell carcinoma

## Abstract

**Key Clinical Message:**

Portal vein thrombosis (PVT) can indicate underlying conditions, such as malignancy. A case of PVT was later diagnosed as renal cell carcinoma (RCC), highlighting the need to consider cancer in PVT cases and ensure a comprehensive evaluation.

**Abstract:**

Portal vein thrombosis (PVT) is a rare complication that can arise from various underlying conditions. We present a case report of a patient initially presenting with PVT, which was later diagnosed as renal cell carcinoma (RCC). This case highlights the importance of considering malignancy as a potential cause of PVT and the need for a comprehensive evaluation.

## 1 INTRODUCTION

Renal cell carcinoma (RCC) is the most common type of kidney cancer, typically presenting symptoms related to the urinary tract or metastatic spread.^1^ However, RCC can occasionally manifest with atypical signs and symptoms, leading to diagnostic challenges. Portal vein thrombosis (PVT) is a rare complication of RCC,^2^ and its presence as the initial presentation is even rarer. In this case report, we discuss a unique case in which PVT served as the presenting sign of RCC, highlighting the importance of considering RCC in the differential diagnosis of patients with PVT.

## 2 CASE HISTORY/EXAMINATION

A 50‐year‐old female patient presented to the emergency department with a chief complaint of epigastric pain lasting for 5 days. The pain began gradually and was rated as 5 out of 10 in intensity. It was constant and did not radiate to the back or shoulder. The patient reported experiencing associated nausea but no vomiting, abdominal distension, diarrhea, or constipation. Furthermore, the patient denied any dysuria, frequency, or oliguria history. There were no signs of fever, weight loss, or anorexia. Additionally, there was no evidence to suggest any connective tissue diseases in the patient's medical history. Notably, the patient had a previous medical history of a left breast fibroadenoma surgically removed 10 years ago. There was no history of smoking or alcohol consumption and no history of cirrhosis. The patient was not taking any medications at home.

A physical examination revealed no significant abnormalities except mild tenderness in the epigastric region.

## 3 METHODS (DIFFERENTIAL DIAGNOSIS, INVESTIGATIONS, AND TREATMENT)

**FIGURE 1:**
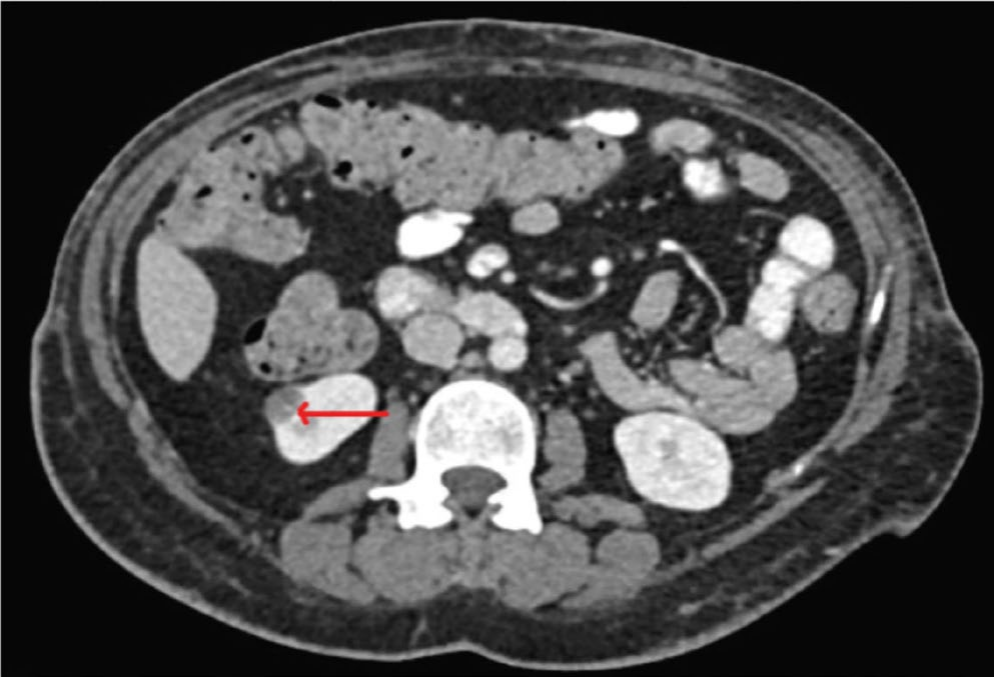
A CT scan of the abdomen with contrast showing a right renal complex cystic lesion.

**FIGURE 2:**
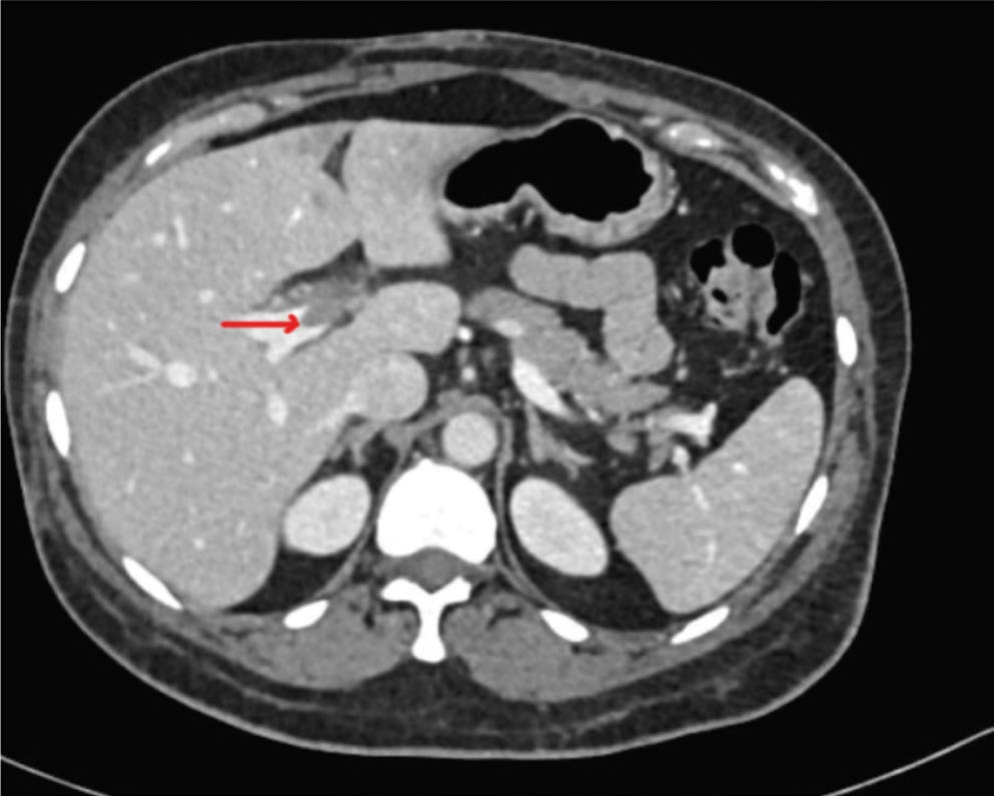
A CT scan of the abdomen with contrast showing portal vein thrombosis.

The patient underwent basic blood tests and imaging studies to further investigate her condition. A CT scan of the abdomen with contrast (refer to Figures 1 and 2) revealed the presence of left portal vein thrombosis and a complex cystic lesion in the right kidney. To explore potential causes of portal vein thrombosis, additional investigations were conducted to rule out thrombophilia syndromes and autoimmune diseases, and the results were negative (refer to Table 1). Subsequently, the patient was initiated on anticoagulation therapy.

**TABLE 1:** Descriptions of common blood tests including coagulation profile.

Laboratory test	Result	Normal value
White blood cell count	7 × 10^3^/μL	(4–10) × 10^3^/μL
Hemoglobulin	13.2 g/dL	(13–17) g/dL
Platelet	342 × 10^3^/μL	(150–400) × 10^3^/μL
Urea	4.2 mmol/L	(3.2–7.4) mmol/L
Creatinine	61 umol/L	(64–110) umol/L
HbA1C %	5.8%	4.8%–5.9%
C‐reactive protein	63 mg/L	(0–5) mg/L
Alanine aminotransferase	60 U/L	(0–41 U/L)
Aspartate aminotransferase	38 U/L	(0–40 U/L)
Alkaline phosphatase	95 U/L	(40–129 U/L)
D‐Dimer	0.43 mg/L	0.49–0.00
Prothrombin time	10.0 s	11.8–9.7
INR	0.9	
APTT	27.1 s	31.2–24.6
Fibrinogen	5.83 g/L	4.20–1.70
Protein S activity	106.0%	126.0%–56.1%
Protein C activity	134.7%	70%–140%
Antithrombin activity	93.7%	112.0%–79.4%
Homocysteine plasma	10.4 umol/L	0.0–15.0
Factor v laden	Negative	
ANA CTD	Negative	
Rheumatoid factor	10 IU/mL	0–14 IU/mL
Anticardiolipin Ab IgG	Negative	
Anticardiolipin Ab IgM	Negative	
Anti B2 glycoprotein IgG	Negative	
Anti B2 glycoprotein IgM	Negative	
Lupus anticoagulant	Negative	

**FIGURE 3:**
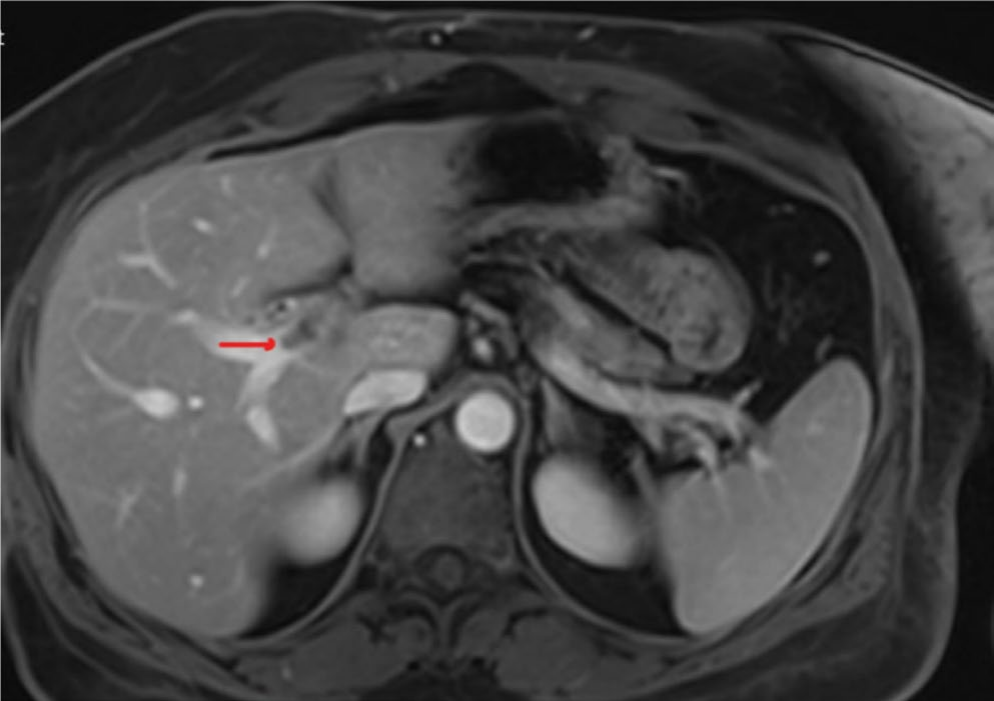
MRI scan of the abdomen showing the left renal vein thrombosis.

**FIGURE 4:**
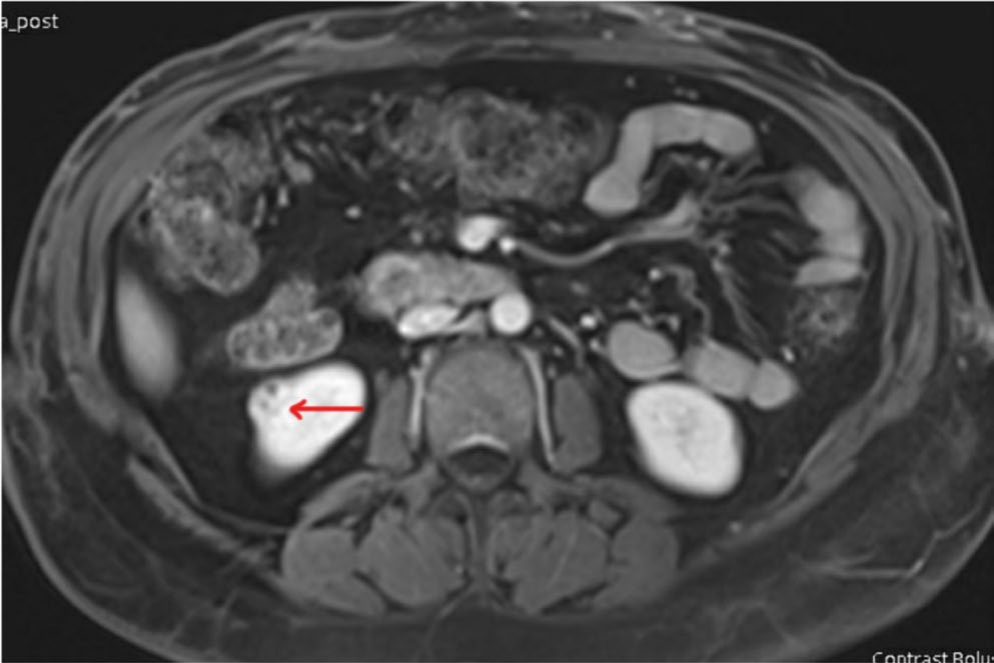
MRI scan of the abdomen with contrast showing right renal mass suggestive of renal cell carcinoma.

An MRI scan of the abdomen with contrast was then performed, and the results indicated that the renal cyst exhibited MRI features consistent with renal cell carcinoma (refer to Figures 3 and 4). Consequently, the portal vein thrombosis was considered secondary to a possible renal cell carcinoma.

## 4 CONCLUSION AND RESULTS (OUTCOME AND FOLLOW‐UP)

The patient was discharged with a prescription for rivaroxaban and scheduled for a follow‐up with the uro‐oncology team. Although a partial nephrectomy was initially offered as a treatment option, the patient expressed reluctance toward surgery and opted for active surveillance. However, later on, the patient traveled to the United States, where she underwent a robotic‐assisted partial nephrectomy. A tissue biopsy subsequently confirmed the presence of clear‐cell renal cell carcinoma.

## 5 DISCUSSION

We are reporting a case of RCC presented with portal vein thrombosis.

Portal vein thrombosis is a relatively rare condition that can occur due to various etiologies, including cirrhosis, hypercoagulable states, infection, and malignancy. RCC‐associated PVT is an uncommon phenomenon, accounting for approximately 4%–10% of cases.^3^ However, it is crucial to consider malignancies, especially RCC, as an underlying cause, even in the absence of typical risk factors. RCC is known to exhibit unique characteristics such as tumor thrombus formation, which can extend into the renal vein, inferior vena cava, and even the hepatic veins.^2^

The mechanism behind PVT development in RCC involves the tumor's direct invasion into the renal vein, subsequently propagating into the portal vein. The underlying mechanisms that promote thrombosis in RCC remain poorly understood but are likely multifactorial, involving tumor‐derived procoagulant factors, platelet activation, and endothelial dysfunction.^4^

In this case, the patient's initial abdominal pain symptoms and the presence of PVT raised suspicion for an underlying liver pathology. However, the subsequent imaging studies, particularly the contrast‐enhanced CT scan, identified renal mass as the primary cause. It is important to note that RCC‐associated PVT can present with nonspecific symptoms, which may mimic other etiologies, further complicating the diagnosis.

The diagnosis of RCC as the underlying cause of PVT can be challenging due to the lack of specific symptoms and the rarity of this presentation. Patients with RCC‐associated PVT often present with nonspecific symptoms related to the liver and the gastrointestinal tract.^5^ These symptoms may mimic other conditions such as liver cirrhosis, hepatocellular carcinoma, or gastrointestinal malignancies. Consequently, the diagnosis of RCC can be delayed, leading to a poorer prognosis. Therefore, it is crucial to consider RCC as a potential cause in patients presenting with PVT, especially when there are no other evident etiologies.

Imaging studies play a vital role in diagnosing RCC‐associated PVT. Abdominal ultrasound, CT scan, and magnetic resonance imaging (MRI) can detect both the renal mass and the thrombus within the portal vein. These imaging modalities help determine the extent of the thrombus, assess the involvement of adjacent structures, and aid in surgical planning.^2^

The management of RCC‐associated PVT typically involves a multidisciplinary approach. Surgical resection remains the gold standard treatment for localized RCC, often including removing the tumor thrombus.^6^ Systemic therapy with targeted agents or immunotherapy may be considered in cases of metastatic disease or unresectable tumors. Anticoagulation therapy may also be initiated to prevent further clot propagation and manage thrombotic complications.

Prognosis in cases of RCC‐associated PVT depends on several factors, including the stage of the tumor, the presence of distant metastasis, and the response to treatment. Generally, patients with localized disease and an early diagnosis have a better prognosis compared to those with advanced disease. However, the presence of PVT itself is considered a poor prognostic factor, highlighting the need for prompt recognition and treatment.^2^

Our case is the first case reported as localized RCC with concomitant PVT without other thrombotic events or risk factors. It is interesting as portal thrombosis rarely presents as the initial clue of RCC in the absence of any other risk factor, in almost all cases, the RCC is discovered first or recurrence of RCC after nephrectomy in the cause.^5^ It is reasonable as well to look for PVT as one of the complications or paraneoplastic syndromes in RCC patients.

## 6 CONCLUSION

This case report underscores the importance of considering malignancy, particularly renal cell carcinoma, as an underlying cause of portal vein thrombosis. Clinicians should maintain a high index of suspicion for RCC when evaluating patients presenting with PVT, especially in the absence of typical risk factors. Prompt diagnosis and appropriate management are essential to improve patient outcomes and prevent further complications associated with RCC and PVT.

## AUTHOR CONTRIBUTIONS

**Raad Alhaj Tahtouh:** Formal analysis; supervision; writing – original draft. **Wisam Alwassiti:** Formal analysis; writing – original draft; writing – review and editing. **Raidah Sawan:** Investigation; writing – original draft; writing – review and editing. **Ahmad Ali:** Investigation; writing – original draft; writing – review and editing. **Khaled Alkhodari:** Formal analysis; supervision. **Khalid Azhar:** Investigation; resources; supervision.

## FUNDING INFORMATION

Abhath funded this case report at Hamad Medical Corporation.

## CONSENT

Written informed consent was obtained from the patient to publish this report in accordance with the journal's patient consent policy.

## Data Availability

Data are openly available in a public repository that issues datasets with DOIs.
